# Eye, Ear, Nose and Throat

**Published:** 1893-05

**Authors:** 


### EYE, EAR, NOSE AND THROAT.

EDITED BY DR. ARTHUR G. HOBBS, ASSISTED BY DR. GEORGE BROWN.

### CHRONIC SUPPURATIVE OTITIS MEDIA OF TUBERCULAR ORIGIN.

Dr. A. E. Davis says the prognosis in these cases is bad. The local treatment he uses in all uncomplicated cases of chronic suppurative otitis media is a combination of the "wet" and "dry" treatments. That is:

1. Thoroughly cleanse the ear with a warm saturated solution of boracic acid.

2. Drop into the meatus and tympanum a few drops of peroxide of hydrogen.

3. Dry out the tympanum with probe and cotton, remove all crusts and scabs still remaining, and search for dead bone, or any signs that may lead into the mastoid antrum.

4. Blow into the meatus and tympanum a powder composed of

℞. Aceto-tartrate aluminum, ʒii.

Acid boric, ʒiv.

M. Ft. pulver.

5. Allow this to remain in the ear until the next day, when it is to be washed out thoroughly, and the whole process of the preceding day repeated. This is continued daily, or the powder left out a day or two if too irritating.

This treatment in my hands has checked the most stubborn cases of the ordinary forms of chronic suppurative otitis media, some of which had resisted all other treatment. That this failed in the two tubercular cases that I have reported in this article shows that its use is limited. In one of these cases it could not be used at all.—*The Post Graduate, April,* 1893.

---

### WHEN TO ENUCLEATE AN EYE.

The indications for enucleation of an eye are:

1. The presence in the eye of a malignant new growth, as glioma, sarcoma or tuberculosis. This indication is imperative, no matter how much vision the eye retains.

2. The presence in the eye of a foreign body with iridocyclitis. If the injury be recent and the inflammatory process still active, and the patient cannot remain under observation, an eye with anything less than thoroughly useful vision should be sacrificed.

3. The presence of a foreign body in a blind eye.

4. Blindness with diminished tension of the eyeball, following perforation either by traumatism or corneal ulcer; most urgent after traumatic perforation of the exposed portion of the sclera.

5. Blindness the result of iridochoroiditis without perforation of the eyeball, if the patient cannot remain under observation.

6. Sympathetic inflammation, provided the exciting eye does not possess vision sufficiently good to be weighed against the chances of the sympathizing eye.

7. The actual presence of sympathetic irritation; not the risk of it, unless the patient is likely to be out of the reach of surgical aid.

8. Persistent pain in a blind eye, sufficient to annoy the possessor or tempt him to the use of analgesic drugs.

9. Serious disfigurement of a blind eye, even if free from pain or risk of causing sympathetic disease.—*Dr. Edward Jackson, in the Philadelphia Polyclinic, April*, 1893.

---

### IRITIS.

Dr. Arthur D. Mansfield distinguishes three distinct forms of iritis, viz.: (1) Simple iritis; (2) plastic iritis; (3) suppurative iritis.

The second form includes (a) syphilitic iritis, (b) rheumatic iritis, (c) traumatic iritis.

He sums up the treatment of all forms as atropia first; if not borne well substitutes hyoscyamine, duboisine, or homatropine. Should adhesions have formed recommends an ointment of

℞. Atropia sulph., gr. v.

Vaseline, ʒi.

M. Ft. unguent.

Sig.—Apply every hour for a few hours.

For relief of pain, heat applied by immersing eye in cup full of hot water, and then apply hot dry cloths. Salicylate of soda, ten to fifteen grains every three hours, will sometimes relieve the pains considerably. A *dernier ressort* is paracentesis of anterior chamber. In the syphilitic form push mercury and iodide of potash to the full limit. His usual method is to give one-fourth grain protoiodide of mercury in an excess of iodide of potassium, and increase the dose until the disease shows signs of abating.

In rheumatic iritis he finds salicylate of soda and general alkaline treatment to give as good results as any other.—*The Medical and Surgical Reporter, March* 18, 1893.

---

### SUBCONJUNCTIVAL APPLICATION OF COCAINE.

Dr. Kaller (*New York Medical Journal,* January 7, 1893) says First I instill a few drops of a four per cent. solution and wait several minutes, after which the instillation is repeated. Now I insert the speculum and, by means of a sterilized hypodermic syringe, inject a few drops of a two per cent. solution of cocaine under the conjunctiva, next to that part of the cornea where I intend to make the section. This will be the upper part in most cases. The solution has been sterilized previously by boiling it, and the hypodermic syringe by rinsing with alcohol and then with a two per cent. carbolic acid solution. After the injection the speculum is removed, and one has to wait from five to ten minutes for the artificial œdema at the place of injection to subside, as it possibly would be in the way of the knife. If it is slow to disappear, gentle rubbing will hasten it. The anæsthesia thus attained is complete, and will contribute to diminish that percentage of prolapse of the iris that still adheres to our statistics of cataract extraction.—*Medical Record.*

---

A Washington dispatch to a New York daily refers to one busy physician at the capital who had four hundred new patients during the forty-eight hours succeeding the inauguration ceremonies. The poor popular physician is also said to have kept on his clothes during the entire time of continuous attendance upon the poor unfortunates, and neither ate, drank, nor slept during that time. Too much, doctor!—*Medical Record.*

That's nothing. We have a "poor popular physician" in Atlanta who said, not long ago, that he hadn't had time to take off his shoes in two weeks. Next!

